# The Impact of Intradialytic Cognitive and Physical Training Program on the Physical and Cognitive Abilities in End-Stage Kidney Disease Patients: A Randomized Clinical Controlled Trial

**DOI:** 10.3390/brainsci13081228

**Published:** 2023-08-21

**Authors:** Aljaž Kren, Špela Bogataj

**Affiliations:** 1 Faculty of Health Sciences, University of Novo Mesto, 8000 Novo Mesto, Slovenia; aljaz.kren@fsp.uni-lj.si; 2 Department of Nephrology, University Medical Centre Ljubljana, 1000 Ljubljana, Slovenia

**Keywords:** hemodialysis patients, cognitive function, cognitive training, physical exercise, end-stage kidney disease

## Abstract

Background: Hemodialysis (HD) patients have lower cognitive functioning and reduced physical fitness than age-matched healthy individuals. Clinicians typically do not recognize the declining cognitive performance in these patients; therefore, cognitive impairment is greatly underestimated and not appropriately treated. This study aimed to evaluate the impact on cognitive function of combining cognitive training with physical exercise and physical performance in HD patients. Methods: Using a randomized, single-blinded control design, forty-four HD patients were recruited and randomly assigned to either an intradialytic physical exercise and cognitive training program (EXP group; *n* = 22; 54% male; 65.7 ± 9.7 years; 77.1 ± 21.9 kg; body mass index 26.8 ± 6.0) or a standard care control group (CON group; *n* = 21; 77% male; 67.2 ± 12.5 years; 74.2 ± 14.3 kg; body mass index 25.9 ± 3.8). The EXP group performed intradialytic cycling and cognitive training three days per week for 12 weeks. Study outcomes were assessed by the Symbol Digit Modalities Test (SDMT), Montreal Cognitive Assessment (MoCA), 10-repetition sit-to-stand test (10-STS), handgrip strength test (HGS), and stork balance test. Results: The results showed a significant time*group interaction effect for SDMT (*p* < 0.001; η^2^ = 0.267) and MoCA (*p* < 0.001; η^2^ = 0.266). Moreover, no significant interaction was observed for 10-STS, HGS, and stork balance test (*p* > 0.05). Conclusions: Our findings suggest that incorporating intradialytic cognitive and physical exercise training could help to improve the functional status of HD patients. The innovative, nonpharmacological, bimodal intervention is cost-effective, safe, and easy to implement during the intradialytic period and offers a potential impact on patients’ quality of life and well-being.

## 1. Introduction

Chronic kidney disease (CKD) has become a major contributor to mortality and morbidity in the twenty-first century, with an estimated 1.2 million global deaths and 35.8 million disabilities attributed to the condition in 2017 [1]. In fact, projections suggest that CKD could rise from the sixteenth to the fifth leading cause of death, with mortality rates expected to increase from 2.2 to 4.0 million by 2040 [2,3]. CKD patients treated with hemodialysis (HD) are affected in multiple ways. They are prone to a chronic catabolic state and negative nitrogen balance, further deteriorating body composition towards sarcopenia or sarcopenic obesity [4]. Age, renal replacement therapy, inadequate diet, and physical inactivity can lead patients to physical, mental, and social frailty, severely worsening their quality of life and survival [5].

Mild cognitive impairment becomes more prevalent with age. It is even more pronounced in individuals with CKD because of potentially impaired endothelial function, impaired blood-brain barrier permeability, increased cerebral microhemorrhage burden, increased cerebral blood flow, impaired cerebral autoregulation, impaired cerebrovascular reactivity, and increased arterial stiffness [6]. Many studies have found a significant deterioration in cognitive function among HD patients, with a prevalence of up to 87% [7,8,9,10]. CKD patients are also prone to developing cardiovascular disease and stroke [11]. Patients undergoing HD have impaired executive functions, and their mental and cognitive abilities are regulated by goal-directed behavior and problem-solving [12]. These weakened cognitive abilities and executive functions can lead to problems with adherence to treatment regimens [9], more frequent visits to emergency departments, difficulties adhering to prescribed therapy, higher mortality, and worsening of functional abilities, quality of life, maintaining independence, and self-care. [13,14,15,16]. It appears that cognitive impairment is frequently associated with unfavorable outcomes, indicating that an intervention targeting cognitive decline could potentially enhance the quality of life of patients undergoing HD [17,18].

To date, establishing evidence-based treatments for cognitive impairment in HD patients has proven challenging. There are numerous obstacles to devising effective therapies for cognitive impairment in this cohort. One primary challenge is that cognitive impairment typically results from multifactorial causes, which makes it unlikely for a single therapeutic agent to effectively counteract the diverse pathological mechanisms at play [19]. The existing evidence is insufficient to endorse the idea that cognitive training can mitigate or improve cognitive decline in this population [18].

Sedentary behavior is very common among HD patients, and nearly one-third of patients rarely or never engage in physical activity [20,21,22]. Recent studies highlight the increasingly important role of physical activity in addressing a range of cognitive impairments [23]. A systematic review of studies that have examined the impact of physical exercise on cognition in older adults has provided sufficient evidence that physical exercise positively affects cognitive abilities (cognitive efficiency, global cognition, executive functions, learning, memory, and language) in the older population [24]. Physical exercise slows down age-related atrophy of the frontal cortex, which is responsible for executive functions [25]. People with good physical fitness can tolerate a greater neuropathological burden without suffering cognitive impairment [26]. Physical activity maintains the neural network through neuroplasticity, brain perfusion, and neurogenesis [25].

Cognitive training and exercise over a three-month period improved executive function in older adults and were more effective than cognitive training or exercise alone [27,28]. In addition, the pilot study on HD patients demonstrated that cognitive training and physical exercise separately prevented cognitive decline [29]. A growing number of studies on exercise training in the HD population show improvements in quality of life [30,31], body composition [32], inflammation [33], dialysis symptoms [34,35], and cognitive function [36,37]. Despite the previously mentioned positive impacts of physical exercise, there is a lack of randomized controlled trials that explore its effects on cognitive performance in HD patients.

Additionally, an evident gap exists regarding the application and comprehensive understanding of combined physical exercise and cognitive training interventions in HD patients. Most research in this area has only examined the effects of physical exercise interventions and limited exploration of the combined effects of physical exercise with cognitive training. Given these findings, it seems promising to combine physical exercise and cognitive training to improve the functional status of HD patients, both cognitively and physically. The HD procedure provides a unique opportunity to conduct such combined interventions.

The purpose of this study is to investigate the effects of a program that integrates cognitive training and physical exercise during HD on the cognitive and physical function of HD patients. This study aims to demonstrate the efficacy of this program in addressing the prevalent challenges of cognitive impairment and reduced physical fitness often faced by HD patients. With the high prevalence of cognitive and physical impairments in this patient group and the current lack of effective non-pharmacological interventions, our study seeks to explore an innovative, cost-effective, and safe intervention that can be conveniently implemented during intradialytic sessions. The primary hypothesis of this study is that HD patients subject to a combined program of intradialytic cognitive training and physical exercise would exhibit significant improvements in their cognitive and physical function compared to the control group receiving standard care.

## 2. Materials and Methods

### 2.1. Participants

We conducted a randomized, controlled interventional trial examining the effects of intradialytic cognitive training in combination with intradialytic physical exercise in a population of HD patients. Seventy-two HD patients were approached in the HD unit of the University Medical Centre in Ljubljana, Slovenia.

The inclusion criteria were the following: participants of any gender over 18 years old (with no upper age limit) had to have undergone HD replacement therapy over three months, be in stable medical condition, and be able to walk independently. Exclusion criteria included were active malignant or infectious disease, uncontrolled arterial hypertension, angina pectoris of Canadian Cardiovascular Society grade 2–4, New York Heart Association heart failure grade of 3 or 4, severe cognitive impairment and dementia, history of limb amputation, and any other condition that could cause the patient to being clinically unstable.

Withdrawal criteria comprised any intercurrent illness or trauma that would prevent the patient from continuing the intervention for a period longer than 14 days; the occurrence of an acute illness lasting longer than three weeks or ending less than three weeks before the end of the study; diagnosis of a malignant disease throughout the research; cerebrovascular or other cardiovascular events (new-onset angina pectoris, myocardial infarction, symptomatic peripheral arterial obliterative disease, heart failure hospitalization); and withdrawal of consent to participate.

Participant eligibility in our study was not limited by factors such as medications, drug interventions, blood pressure, other metabolic disorders, physical fitness level, Equivalents of Task (METs), or Body Mass Index (BMI) category.

National Medical Ethics Committee approval (Ministry of Health, Republic of Slovenia, approval document number KME 0120-474/2021/10) and written informed consent were obtained in all cases. The study was conducted in accordance with the Helsinki-Tokyo Declaration. The clinical trial was registered with ClinicalTrials.gov (NCT05150444).

### 2.2. Outcome Measures

The primary endpoints were Montreal Cognitive Assessment (MoCA) and Symbol Digit Modalities Test (SDMT). The MoCA test is a 30-point, 10-min test. It assesses short-term memory through a five-point recall task; visuospatial abilities with a clock-drawing task (three points) and a cube copy (one point); and multiple executive functions, such as alternation (one point), phonemic fluency (one point), and verbal abstraction (two points). Attention, concentration, and working memory are evaluated (six points), language through naming, repetition, and fluency tasks (eight points), and orientation to time and place (six points). A score of 25 or below indicates impairment [38]. SDMT assesses key neurocognitive functions that underlie many substitution tasks, including attention, visual scanning, and motor speed [39]. In this test, a coding key includes nine unique abstract symbols, each of which is paired with a number from 1 to 9. The individual taking the test must quickly scan the key and write down the number that matches each symbol. Their performance is measured by the number of correct substitutions made within a 90-s time frame. In the test’s written format, the subject is responsible for filling in the numbers that align with the corresponding symbols. Scores for the test range from 0 to 110 based on the number of items accurately coded within the 90-s limit [40].

The secondary endpoints were handgrip strength test (HGS), assessed with a calibrated hydraulic hand dynamometer (Jamar, Patterson Medical, Warrenville, IL, USA), which measures maximum muscle strength [41]; balance, assessed by the Stork test on a foam pad (Airex, Sins, Switzerland), which measures an individual’s balance and stability on one leg [42]; and the 10-repetition sit-to-stand test (10-STS), which measures lower limb muscle strength and the time it takes an individual to stand up and sit down ten times from a seated position [43]. All tests were executed in a fixed order, as previously described, to minimize patient fatigue. Before the tests, we measured height and weight. It took approximately 30 min for a patient to complete all outcome assessments (including rest periods).

### 2.3. Procedure

The testing was conducted on days when the patients did not receive dialysis treatment [44]. Patients who underwent dialysis on a Monday-Wednesday-Friday schedule were tested on Saturdays, while those who received dialysis on a Tuesday-Thursday-Saturday schedule were tested on Fridays. The outcomes were evaluated at two different time points: before the intervention and after 12 weeks, using the same assessors who were blinded to the treatment allocation [45]. However, the patients and in-center dialysis care providers were not blinded to the intervention. After baseline testing, the patients were randomized using an open-source computer program Research Randomizer 4.0 [46] and allocated in a 1:1 ratio to the cognitive and exercise training group (EXP) or the control group with standard HD care (CON). The EXP group performed aerobic exercise during the first half of the dialysis treatment (three times a week for 12 weeks) for up to 60 min on a customized ergometer (Lemco bed bike). They started with a 3-min warm-up, then the resistance was applied to each individual according to the rate of perceived exertion of fourth to fifth grade on a 10-grade Borg scale [44]. Intradialytic cycling was prescribed and monitored by a registered nurse or kinesiologist. After a break, they were given tablets in order to play “ brain games” on a CogniFit platform (30 min). The difficulty of the “brain exercises” adapted automatically to the patient’s skills as they practiced and trained. The targeted cognitive domains were memory, reasoning, coordination, and attention, with their subcategories.

**Figure 1:**
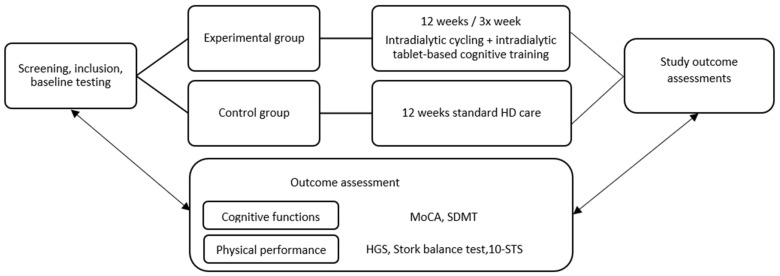
Schematic design of the experimental protocol.

Graphically we presented the study design in Figure 1.

### 2.4. Statistics

SPSS 24.0 (SPSS, Inc., Chicago, IL, USA) software was used for all calculations. All data was presented with a mean ± standard deviation. Normality was confirmed by using the Shapiro-Wilk test, and Levene’s test was used to check the assumption of equal variances. The main effects were analyzed using a mixed general linear model (GLM), which considered the groups (EXP and CON) and time (baseline and after 12 weeks) as factors. Additionally, the degree of effect was determined for dependent variables using partial eta-squared (η^2^). Partial eta squared readings of 0.02, 0.13, and 0.33 were rated small, moderate, and high [47]. The statistical significance for the analysis conducted in this study was set at a *p*-value < 0.05. We employed two-tailed tests for all statistical analyses to account for potential effects in both directions.

The sample size was calculated based on the results of our previous study [44] using G*Power software version 3.1.9.7. The calculation was based on the results of the 10-STS test before the intervention and after the intervention. The probability of alpha error was set at 0.05, the probability of 1-beta error at 0.95, and the effect size was taken from the aforementioned study (1.21). The calculated sample size was 38 subjects. If we consider an expected 10% dropout rate, it is necessary to randomize 42 subjects.

## 3. Results

### Participants’ General Characteristics

**Figure 2:**
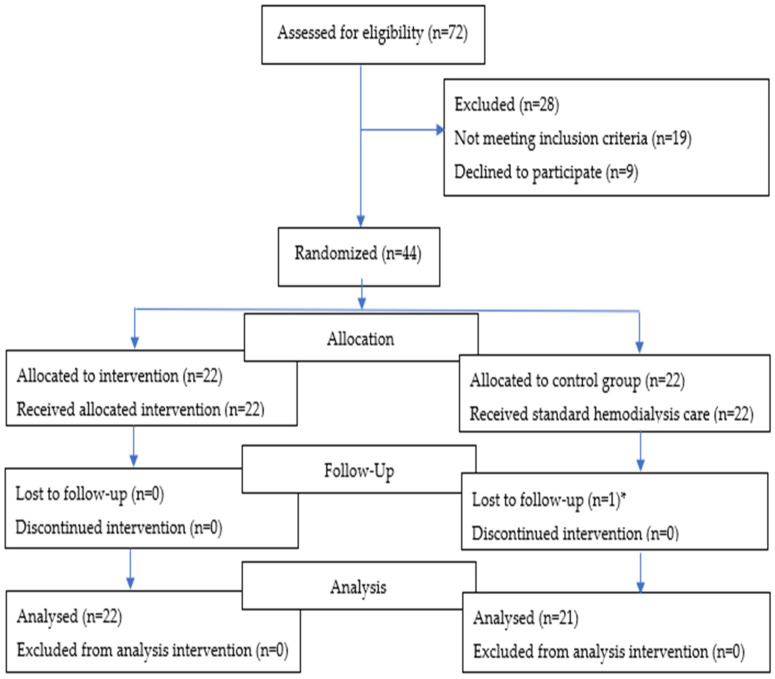
CONSORT (Consolidated Standards of Reporting Trials) diagram. * Lost to follow up in control group; infection with Carbapenem-resistant Enterobacteriaceae (*n* = 1).

The study took place from October 2022 to January 2023. Out of 72, 44 patients were randomized, and 43 completed the study (Figure 2).

Overall, Table 1 shows that the experimental and control groups were fairly similar in terms of demographic and clinical characteristics, with the exception of a slightly higher percentage of male participants in the control group.

Table 2 shows the adherence rates for the EXP group in both intradialytic cycling and cognitive training. In the case of intradialytic cycling, the adherence rate was 79.9%, indicating that the participants in the experimental group completed approximately 80% of the prescribed cycling sessions. Additionally, the average cycling time per session was 37.6 min.

Regarding cognitive training, the adherence rate was 84.2%, indicating that the participants in the experimental group completed approximately 84.0% of the prescribed cognitive training sessions. Moreover, the average cognitive training time per session was 30 min.

The results from Table 3, analyzed using a two-way analysis of variance for repeated measures, we did not identify statistically significant interactions between the factors of time and group for any of the variables (*p* > 0.05; η^2^ = 0.035–0.091). We also did not identify a statistically significant effect of the group (*p* > 0.05; η^2^ = 0.001–0.069). For the HGS variable (*p* < 0.001; η^2^ = 0.240) and the Stork test, we identified a statistically significant effect of time (*p* = 0.038; η^2^ = 0.101), while we did not identify a statistically significant effect of time for the 10 STS variable (*p* = 0.300; η^2^ = 0.026).

The results from Table 4 show that the experimental group improved their MoCA test score by 2.3 and their SDMT test by 1.0. The control group experienced a decrease in both their MoCA and SDMT test scores, recording values of 2.6 and 1.0, respectively.

Using a two-way analysis of variance for repeated measures, we identified statistically significant interactions between the factors of time and group for the variables of the MoCA test and the SDMT test (*p* < 0.001; η^2^ = 0.266–0.267). For the variable MoCA test, we identified a statistically significant effect of the group (*p* < 0.001; η^2^ = 0.266) and time (*p* < 0.001; η^2^ = 0.434). For the variable SDMT test, we did not identify a statistically significant effect of the group (*p* = 0.919; η^2^ = < 0.001) and time (*p* = 0.051; η^2^ = 0.090).

The study found (Table 5) that the subjects experienced mainly isolated hypotension with no major cardiac events. Throughout the execution period of the study, several practical and logistical observations came to our attention. The primary reasons for participants skipping the cycling sessions and cognitive training were fatigue, high blood pressure, hypotension, the presence of vascular access-related hematoma, joint pain, infection, and holidays.

## 4. Discussion

Our randomized controlled trial aimed to evaluate the impact of a 12-week combined intradialytic cognitive training and physical exercise intervention on cognitive and physical performance in HD patients. Outcomes included the MoCA test, SDMT test, 10-STS, HGS, and Stork test. Results demonstrated a significant improvement in the experimental group’s MoCA and SDMT test scores compared to the control group. Although both groups experienced changes in physical performance measures, the differences were not statistically significant. Intradialytic cognitive and physical training was well-received and tolerated by patients, with no significant adverse events recorded. This response characterizes the intervention as a safe, effective, and easy-to-implement method.

Cognitive decline can manifest on a continuum from mild cognitive impairment to clinically relevant dementia when interference with daily life and independency is present. Measurement of cognitive function is not currently part of the physical examination and medical history of CKD patients in Slovenia. As a prodromal stage, mild cognitive impairment should be identified and studied in CKD before irreversible damage is present [48,49]. In HD patients, cognitive impairment is highly prevalent, with over 70% of patients exhibiting impairments in at least one cognitive domain [50]. The prevalence of mild cognitive impairment in our study was 51.1%. It seems inevitable that cognitive impairment will occur in the natural history of HD patients. Whatever the trigger may be, it seems prudent to limit the progression of cognitive impairment in this group of patients. Cognitive impairment is a significant concern among HD patients, with limited data available on effective interventions. The authors Vanderlinden et al. [51] found in their meta-analysis that the average MoCA score among HD patients is 23.42 ± 3.49. The global MoCA score in our study was 24.6 ± 2.8. However, it is generally observed that these patients tend to have lower MoCA scores than the general population (MoCA score 27.4), indicating a higher prevalence of cognitive impairment [38]. The results from a prospective cohort study showed that patients’ cognitive impairment in memory, executive function, and visual-spatial domains ranged from 40% to 50% [52]. The domain with the highest proportion of impairment is attention [12,52]. A meta-analysis of 42 studies covering 3522 participants found that people treated with HD had worse cognition than the general population, particularly in attention/processing speed and memory [53]. A recent meta-analysis of randomized controlled trials showed that intradialytic and interdialytic exercise significantly improve cognitive impairment in patients undergoing HD [54]. Moreover, in a pilot study [29], cognitive and exercise training performed separately were able to prevent a decline in executive functions and psychomotor speed in HD patients.

In our study, we combined intradialytic cycling with cognitive training and observed a significant improvement in MoCA (+2.3 points) and SDMT (+1 point) scores in the experimental group (*p* < 0.001) compared to the control group. A potential explanation for the successful impact of cognitive and exercise training on cognitive function could be that these strategies strengthen the synaptic connections between neurons and are believed to boost brain plasticity [55,56]. Cognitive training techniques that cover multiple domains are more favored than merely focusing on memory training. Such a multi-faceted method has been linked to a wide array of cognitive function improvements and offers long-term advantages that can be applied to daily tasks even a decade after the intervention [57]. Due to the notable association between weak physical performance and increased mortality in patients with CKD, numerous prospective studies and meta-analyses have been conducted to evaluate the potential benefits of exercise interventions in enhancing patient outcomes [58,59,60,61]. Csuka and McCarty [62] first described the use of the STS test as a measure of lower-extremity strength (force-generating capacity of muscle). The literature results have shown remarkable improvements in STS after long-term intradialytic exercise [63,64]. In the experimental group, we found 1.5 s (6.1%) absolute mean improvement in the 10-STS time. On the other hand, the control group showed a slight increase in their 10-STS times, going from 25.4 s at baseline to 25.5 s after 12 weeks, indicating a slight decline in performance (*p* = 0.227). Our results were lower than those found in a randomized controlled trial study [44], which found a statistically significant improvement in the 10-STS (−4.5 ± 1.9 s, 95% CI −8.4 to −0.7; *p* = 0.021) and the prospective observational cohort study [65] (35.8 ± 17.7 vs. 31.8 ± 15.3 s). On the other side, results from Pavone et al. [32] study did not show a significant improvement within the experimental group. On the contrary, the 10-STS performance had significantly deteriorated in the exercise group and considerably improved in the controls. Moreover, intergroup results for 10-STS showed that the control group improved in time compared to the experimental group.

Over the past ten years, a considerable amount of evidence has indicated the predictive value of HGS as a marker for muscle function and the likelihood of mortality and morbidity outcomes among individuals with CKD [66,67,68]. Our results suggest that the intervention did not significantly impact (*p* = 0.05) HGS test scores, while the control group showed a larger decrease after 12 weeks. Studies have shown that regular resistance training can significantly improve HGS in different populations, including older adults and patients with chronic diseases. Therefore, it is reasonable to assume that a program of endurance-resistance training could have similar beneficial effects on HGS [44,69,70], but our program did not, as it included aerobic training only.

In patients receiving long-term dialysis, there is a notable decrease in physiological reserves [71]. This treatment also leads to nutrient loss in the body, heightening the risk of falls. Particularly for those undergoing HD who are at an elevated risk for falls and fractures, there may be impaired balance and flexibility, followed by a deterioration in lower limb functions [72,73,74]. Upon comparing the experimental and control groups, it was observed that the experimental group demonstrated a more considerable improvement in their Stork test scores (1.3 s) after 12 weeks than the control group did (0.2 s), but the difference was not statistically significant (*p* = 0.089). Participating in endurance-resistance training can help improve an individual’s performance in the Stork Test. As endurance-resistance training targets multiple aspects of physical fitness, it can enhance balance, flexibility, and lower limb strength, all of which contribute to better performance in the Stork Test and a reduced risk of falls [75,76,77].

So far, there has been no successful development of a pharmacological drug capable of reversing dementia. Additionally, the potential side effects of medications aimed at alleviating symptoms might not be worth the benefits they provide [78]. Cholinesterase inhibitors and methyl D-aspartate receptor antagonists delay cognitive decline by approximately six months in dementia patients; however, their safety in end-stage renal disease (ESRD) remains unclear. High-dose vitamin B, increased Kt/V, and frequent HD sessions have shown no significant cognitive improvement in HD patients. Conversely, erythropoietin therapy, nocturnal HD, group-based cognitive behavioral intervention, kidney transplantation, and exercise have demonstrated some positive effects on cognition [79,80,81,82,83]. Nevertheless, pharmacological approaches encounter obstacles such as restricted applicability, side effects, and logistical concerns, which call for additional research to determine suitable options for HD patients. In summary, non-pharmacological methods seem more favorable for preventing cognitive decline and, consequently, possibly lowering the incidence of dementia. Our study demonstrated that a combination of cognitive training and physical exercise during dialysis might enhance or at least prevent a decline in cognitive function and physical performance in HD patients. However, more studies are needed to confirm this.

While MoCA and SDMT are validated tools, they do not address all aspects of cognitive function. The psychological assessment of cognitive function could be deepened by a clinical neuropsychological assessment, which is more detailed about the subdomains of cognitive function than the MoCA screening test we used. Likewise, the Stork test, HGS, and 10-STS primarily focus on physical functioning but might miss aspects related to endurance, flexibility, or other relevant functional abilities. The study does not provide information on the long-term effects and sustainability of the intervention.

This research delivers persuasive data that points to the beneficial effects of combining intradialytic cognitive training with physical exercise to enhance cognitive functionality in patients undergoing HD. However, it is essential to continue studying the long-term outcomes of this intervention and identify the most effective methods for its application within a clinical environment.

While the cognitive function saw measurable enhancement, the study did not observe significant changes in physical performance metrics. This suggests that further exploration is needed into the most beneficial exercise methods for those undergoing HD. Furthermore, it would be worthwhile to examine if lengthening the duration of the intervention might result in notable improvements in physical function. Considering the high incidence of cognitive impairment among HD patients, it would be advisable to expand research aimed at the early detection and prevention of cognitive decline in this group, thereby improving their overall quality of life and independence. Our study has several limitations that should be acknowledged. The study’s participants were recruited from only one HD unit at the University Medical Centre in Ljubljana, Slovenia. The single-center nature of the study may limit the generalizability of the results for other HD patient populations in different geographical locations or healthcare settings. Additionally, the strict inclusion and exclusion criteria might have resulted in a cohort that is healthier or has less comorbidity than the general HD population, potentially limiting the study’s generalizability. Our sample had a relatively low mean age. This suggests the possibility that older patients may demonstrate less tolerability to physical exercise interventions, which could limit the scope of our findings. Future investigations should consider including four distinct groups: one dedicated solely to intradialytic cycling, another to cognitive training, a third group receiving both interventions, and a control group provided with standard HD care. Due to the intervention’s nature, it was impossible to blind the investigators and subjects. However, we ensured that those assessing the outcomes were blinded to group allocations. Lastly, we did not measure aspects like nutritional status, VO2 max, and METs. The absence of these parameters in our data collection might have influenced our findings or resulted in unexplained variances. It is worth acknowledging that these measures could offer additional insights into the participants’ overall fitness and metabolic health.

These limitations are offset by various strengths. This study is the most extensive one conducted within the Slovenian context, and it utilized a practical clinical cohort to guarantee the transferability and generalizability of the findings. Additionally, it was one of the pioneering studies to examine the combined impact of a distinctively designed physical exercise and cognitive training program on the functional status of HD patients. Nevertheless, these findings need to be validated through larger, multi-center trials to ascertain the potential improvements in physical performance measurements. Future research could also consider assessing the combined effects of other non-pharmacological interventions, such as dietary alterations, stress management techniques, sleep hygiene practices, and social support systems, in conjunction with cognitive training and exercise. Additional types of exercise interventions might be worth exploring in future studies to enhance physical performance in patients undergoing HD. In our study, we chose intradialytic cycling for its feasibility within the dialysis session. Although it resulted in a trend towards improvement in physical outcomes, it might not have targeted all the necessary aspects of physical fitness required by HD patients. Therefore, a more comprehensive physical exercise program, possibly including resistance, balance, and flexibility exercises, could potentially result in more significant and widespread improvements.

## 5. Conclusions

In summary, this study offers insights into the combined effects of cognitive training and physical exercise on cognitive and physical function in HD patients. The experimental group, which underwent intradialytic cycling along with cognitive training, showed a significant improvement in cognitive function, as evidenced by the increase in scores on the MoCA and SDMT. The method was well-received and tolerated by the patients undergoing HD, with no major adverse events recorded, emphasizing its safety, effectiveness, and feasibility. Our original study demonstrates that this combined physical and cognitive intradialytic training intervention may serve as an effective tool to prevent physical and cognitive decline in the HD population, presenting a promising direction for further exploration and implementation in HD care.

## Figures and Tables

**Table 1:** Demographic and clinical characteristics.

	Experimental Group (*n* = 22)	Control Group (*n* = 21)	All Participants (*n* = 44)
Male sex (%)	54%	77%	66%
Age (years)	65.7 ± 9.7	67.2 ± 12.5	66.5 ± 11.0
Height (cm)	169.6 ± 12.5	171.0 ± 10.5	170.3 ± 11.4
Weight (kg)	77.1 ± 21.9	74.2 ± 14.3	75.6 ± 18.3
BMI (kg/m^2^)	26.8 ± 6.0	24.9 ± 3.8	25.9 ± 5.1
Heart rate (bpm)	72.7 ± 10.4	72.8 ± 15.1	72.8 ± 12.7
Systolic blood pressure (mm Hg)	160 ± 22	151 ± 22	156 ± 22
Diastolic blood pressure (mm Hg)	88 ± 13	85 ± 9	87 ± 11
Fat tissue index (kg/m^2^)	14.6 ± 6.6	12.4 ± 4.0	13.5 ± 5.5

Note: The values are expressed as mean ± SD or in percent. There were no statistically significant differences between the groups. Abbreviations: *n*, number of subjects.

**Table 2:** Adherence to training programs.

	Experimental Group (*n* = 22)
Intradialytic cycling	79.9%
Cycling time (min/session)	37.6
Cognitive training	84.2%
Cognitive training (min/session)	30

Note: The values are presented as mean, and adherence to training programs is defined as the ratio of the total number of completed exercise sessions to the total number of sessions offered/advised. The abbreviation “*n*” refers to the number of subjects.

**Table 3:** Physical performance outcomes throughout the research periods for the experimental and control group.

	Experimental (*n* = 22)		Control (*n* = 21)		Time × Group		
	Baseline	12 Weeks	Baseline	12 Weeks	F	*p*	η^2^
10 STS (s)	24.3 ± 6.6	22.8 ± 6.3	25.4 ± 7.3	25.5 ± 7.3	1.5	0.227	0.035
HGS (kg)	31.3 ± 8.2	30.8 ± 8.8	32.4 ± 9.5	30.6 ± 9.5	4.0	0.051	0.091
Stork test (s)	1.9 ± 1.4	3.2 ± 2.4	2.4 ± 2.2	2.6 ± 3.5	3.0	0.089	0.069

Note: The values are expressed as mean ± SD. There were no statistically significant differences between the groups. η^2^ indicates Partial Eta Squared, *p* < 0.05 indicates significance between group differences compared to the baseline value.

**Table 4:** Cognitive performance outcomes throughout the research periods for the experimental and control group.

	Experimental (*n* = 22)		Control (*n* = 21)		Time × Group		
	Baseline	12 Weeks	Baseline	12 Weeks	F	*p*	η^2^
MoCA test	25.0 ± 2.7	27.3 ± 2.3	24.2 ± 3.0	24.7 ± 3.5	14.8	<0.001	0.266
SDMT test	27.5 ± 11.6	28.5 ± 11.8	27.8 ± 12.4	27.5 ± 12.6	14.9	<0.001	0.267

Note: The values are expressed as mean ± SD. There were no statistically significant differences between the groups. η^2^ indicates Partial Eta Squared, *p* < 0.05 indicates significance between group differences compared to the baseline value.

**Table 5:** Adverse events and specific observations.

Adverse Event	Experimental Group (*n*)	Comments
Hypotension episodes	3	Three patients presented hypotension after a cycling session.
Dyspnea	1	One patient could not perform the cycling session due to dyspnea unrelated to exercise.
Hypertension episodes	3	Three patients could not perform the cycling session due to hypertension unrelated to exercise.
Vascular access hematoma	3	Three patients could not perform the cycling session due to hematoma unrelated to exercise.
Fatigue episodes	7	Seven patients could not perform the cycling session due to fatigue unrelated to exercise.
Joint pain	4	Four patients could not perform the cycling session due to knee or hip pain unrelated to exercise.
Infection	4	Three patients missed a few exercise and cognitive sessions due to COVID-19 infection and one due to influenza infection.
Vacation	2	Two patients missed a few exercise and cognitive sessions due to vacation.
MACE	0	

Abbreviations: *n*, number of events; MACE, major adverse cardiac events.

## Data Availability

The datasets generated during and/or analyzed during the current study are available from the corresponding author upon reasonable request.
